# Dental Notes

**Published:** 1899-07

**Authors:** F. S. Casper

**Affiliations:** Austin, Texas


### Dental Department.

### Dental Notes.

F. S. CASPER, D. D. S., AUSTIN, TEXAS.

### PORCELAIN INLAYS.

The ambition of the progressive dentist at this time seems to be to achieve success in the direction of porcelain inlays. This is a move in the right direction. We can not think of anything more unsightly to the æsthetic eye than a beautiful young lady or a gentleman with the entire row of front teeth filled with gold, having all the appearance of having just emerged from a jeweler's establishment; a walking advertisement, if you please. Of course, we recognize that gold is the ideal filling material, and should always have the precedence over amalgam or any other substance used as a filling. But if an inlay can be attained, which will simulate nature, and meet all requirements, then let us exclaim; "away with your unsightly jewelry work."

Many difficulties are met with in this new field of progress, or rather we should say, old field resurrected, for many have attempted inlay work with doubtful success. For many years our efforts in this direction have often proved gratifying to our patients. Although we were unfitly prepared to accomplish more than partial success, having to grind the inlay from an artificial tooth selected with a view of having color suitable to match the natural organ. This was not always easy to accomplish. Again, our troubles would increase, when we tried to grind the cavity in the tooth to receive the inlay, into proper shape. Many such cavities are on the labial surface of the teeth, and are very sensitive to the touch of an instrument. It must be remembered that caries in a tooth does not leave the cavity it forms in a desirable shape for inlay work, and for this reason mainly have the efforts of many in this direction been abandoned.

While inlay work has not proved satisfactory from the dentist's standpoint, it is because it was never brought to a degree of perfection that has characterized other operations. Our patients seem generally pleased with even a poor excuse for an inlay.

Dr. Jenkins seems to have blazed a way out of this troublesome wilderness. His system consists of a new body, low fusing, sharp, well-defined edges, great strength, and a variety of colors. Then there is a very neat furnace all beautifully gotten up so that inlays can be made and baked in the operating room.

Previously, platinum foil was in use to take impressions of cavities by burnishing the foil to all parts of the cavity. The objection to this was that platinum would stick to the baked body of the inlay. This has to be avoided by the substitution of No. 30 and 40 gold foil.

After taking the impression, the little cup of gold, which is the mould for the inlay body, is embedded in a paste of powdered asbestos, and after sufficiently drying this investment, the body, mixed with absolute alcohol, to the consistency of thick cream, is then put into the matrix and placed in the furnace to be fused. If after heating to fusing point the body shrinks away, more paste should be added until the proper contour is attained. When completed this makes a beautiful piece of work, which is not discernable to the eye.

---

To remove deposits or green stain from the teeth apply dilute tincture of iodine to the stain with a piece of orangewood, and after a short interval use a solution of carbonate of ammonia, which will take off the iodine, and with it the objectionable stain. The teeth should be carefully polished afterward. This is especially good for the destructive "green stain," so prevalent in children's teeth.

---

In reply to several inquiries, we will say, the fountain spittoons alluded to in our June number are not exclusively for the dental profession, but are the ideal thing for the specialist also.

---

### DENTISTS IN THE ARMY AND NAVY.

"The late war has brought to light many improvements, aye, necessities, that are requisite to a successful military campaign. Furthermore, it has proved that it is not best to await the outbreak of actual warfare before adding such necessities. Our regular army was found to be too small; various departments were proportionately so, and there was a total absence of departments that would have added materially to bring our army nearer the state of perfection. The fact that there was no department of dentistry, and that there was great need for that addition, was forcibly impressed upon the mind of Representative Hull, with the resultant clause to his bill, calling for the appointment of one hundred dentists. This bill not having been favorably acted upon as yet, allows for a free discussion of the advisibility of starting this particular branch of the service." —*Dr. Morris I. Schamberg, D. D. S., in International Dental Journal, June, 1899.*

[This measure has been considered many years before the last war, but our wise representatives no doubt look at such suggestion as a measure to supply the soldier with luxuries rather than essentials.—Ed.]

### USEFUL HINTS.

"Small Japanese tooth-picks are excellent for wrapping on cotton to use iodine, aconite and glycerine for the gums, as they are inexpensive and can be thrown away in each case."

"Peroxide of Hydrogen is good for cuts, burns, etc., and will heal in forty-eight hours, and take out all the soreness."

"Bicarbonate of soda will remove all iodine stains, and cause all burning to cease in carbolic acid burns."—*Dr. Lu Ella Cool, San Francisco.*

### TO CLEAN MIXING SLAB.

Use aqua. ammonia; a few drops allowed to remain a short time, will make the cleaning easy.

Gum-mastich dissolved in chloroform makes a varnish which excludes all moisture and acts as a non-conductor.

As a preventive, use nitrate of silver on children's teeth where decay has just commenced. It forms an insoluble compound with lime salt in the tooth structure, thereby preventing the wasting away of these delicate little organs.

